# TIPE polarity proteins are required for mucosal deployment of T lymphocytes and mucosal defense against bacterial infection

**DOI:** 10.1186/s43556-021-00059-8

**Published:** 2021-12-23

**Authors:** Mingyue Li, Mayassa J. Bou-Dargham, Jiyeon Yu, Zienab Etwebi, Honghong Sun, Youhai H. Chen

**Affiliations:** 1 Department of Pathology and Laboratory Medicine, Perelman School of Medicine, University of Pennsylvania, Stellar-Chance Laboratories, 422 Curie Blvd, Philadelphia, PA 19104 USA; 2 The Wistar Institute, Philadelphia, PA 19104 USA; 3 Faculty of Pharmaceutical Sciences, CAS Shenzhen Institute of Advanced Technology, Shenzhen, China

**Keywords:** TNFAIP8, Infection, Mucosal, *Streptococcus pneumoniae*, CCR9, T lymphocytes

## Abstract

Mucosal surfaces are continuously exposed to, and challenged by, numerous commensal and pathogenic organisms. To guard against infections, a majority of the thymus-derived T lymphocytes are deployed at the mucosa. Although chemokines are known to be involved in the mucosal lymphocyte deployment, it is not clear whether lymphocytes enter the mucosa through directed migration or enhanced random migration. Here we report that TIPE (tumor necrosis factor-α-induced protein 8 (TNFAIP8)-like) proteins mediate directed migration of T lymphocytes into lung mucosa, and they are crucial for mucosal immune defense against *Streptococcus pneumoniae* infection. Knockout of both *Tnfaip8* and *Tipe2*, which encode polarity proteins that control the directionality of lymphocyte migration, significantly reduced the numbers of T lymphocytes in the lung of mice. Compared with wild-type mice, *Tnfaip8*^−/−^ *Tipe2*^−/−^ mice also developed more severe infection with more pathogens entering blood circulation upon nasal *Streptococcus pneumoniae* challenge*.* Single-cell RNA-sequencing analysis revealed that TIPE proteins selectively affected mucosal homing of a unique subpopulation of T cells, called “T cells-2”, which expressed high levels of *Ccr9*, *Tcf7*, and *Rag1/2* genes. TNFAIP8 and TIPE2 appeared to have overlapping functions since deficiency in both yielded the strongest phenotype. These data demonstrate that TIPE family of proteins are crucial for lung mucosal immunity. Strategies targeting TIPE proteins may help develop mucosal vaccines or treat inflammatory diseases of the lung.

**Supplementary Information:**

The online version contains supplementary material available at 10.1186/s43556-021-00059-8.

## Introduction

Mucosal surfaces are the first line of immune defense against luminal pathogens [1]. The respiratory and digestive organs have two of the largest mucosal surfaces in the body [2, 3]. In particular, the mucosal surface area in the human lung is as large as a tennis court, approximately 130–180 m^2^ [4]. These mucosal surfaces are the entrance sites for bacterial, viral, and fungal pathogens and provide a route to systemic infections. The host immune system can detect the pathogens and clear the infections while maintaining tolerance to endogenous non-pathogenic microorganisms [5, 6]. In addition to the innate immune system, the pre-existing adaptive immune cells are also able to kill pathogens. For example, in the gut mucosal system, intraepithelial lymphocytes (IELs) are located in the epithelium and can release cytokines quickly upon encountering pathogens [7]. In the lung mucosa, there are similar types of lymphocytes that can respond rapidly to infection. For example, in the lung of naïve mice, pre-existing CD8^+^ T cells enhance resistance to the infection and dissemination of serotype 3 pneumonia [8]. In the lung of humans, pre-existing CD4^+^ T cells produce IL-17 and TNFα after *Streptococcus pneumoniae* infection [9]. However, the specific genes that orchestrate the positioning of these lymphocytes in the mucosa are not fully understood.

During development, lymphocytes are selected in the thymus and their subsequent trafficking to the lung is critical for the host defense response [10, 11]. Chemokines are ligands of G protein-coupled chemokine receptors that regulate leukocyte trafficking [12]. Previous evidence showed that C-C Motif Chemokine Receptor 9 (CCR9), a gut-homing receptor expressed in IELs, is crucial for lymphocyte trafficking. It acts as the receptor for C-C Motif Chemokine Ligand 25 (CCL25) [12]. However, whether CCR9 is employed for the homing of lymphocytes to the lung is largely unknown.

*Streptococcus pneumoniae* (*Sp*) is the leading cause of bacterial pneumonia, especially in elderly people, young children, and immune-deficient patients [13]. It is a gram-positive bacterium that is commonly found on mucosal surfaces [14]. The colonization rates is 5–10% in healthy adults, but can be as high as 50% in infants [14]. *Streptococcus pneumoniae* can cause sinusitis, otitis media and lung pneumonia depending on the local infection sites [15]. Migration and invasion of *Streptococcus pneumoniae* from alveolar spaces into the bloodstream gives rise to a life-threatening systematic infection called septicemia.

The tumor necrosis factor-α-induced protein 8-like (TIPE) family of proteins were initially discovered a decade ago as regulators of inflammation and cancer [16–18]. There are four family members that possess a unique hydrophobic cavity [19, 20], including TNFAIP8, TIPE1 (TNFAIP8L1), TIPE2 (TNFAIP8L2), and TIPE3 (TNFAIP8L3). Among these family members, TIPE2 is preferentially expressed in hematopoietic cells [21, 22]. Our previous research demonstrated the role of TIPE2 and TNFAIP8 in systematic *Listeria monocytogenes* (*L. monocytogenes*) infection induced by intravenous administration of bacteria. We found both TNFAIP8-deficient (TKO) and TIPE2-deficient (T2KO) mice were resistant to *L. monocytogenes* infection. Due to dysregulated RAC1 activation, TNFAIP8 deficiency caused a reduced hepatocyte invasion and an increased apoptosis of infected hepatocytes [22]. The resistance in T2KO mice was related to the increased RAC activation and elevated oxidative burst [23]. Although the previous studies defined the role of TNFAIP8 and TIPE2 in systematic infection, whether they play a role in the mucosal immune system is still unknown. We report here that TIPE proteins (TNFAIP8 and TIPE2) protect mice at mucosal sites from local *Streptococcus pneumoniae* infection by regulating lymphocyte homing, as directed by CCR9.

## Result

### Decreased resident mucosal lymphocyte numbers in TIPE protein-deficient mice at the homeostatic state

**Fig. 1:**
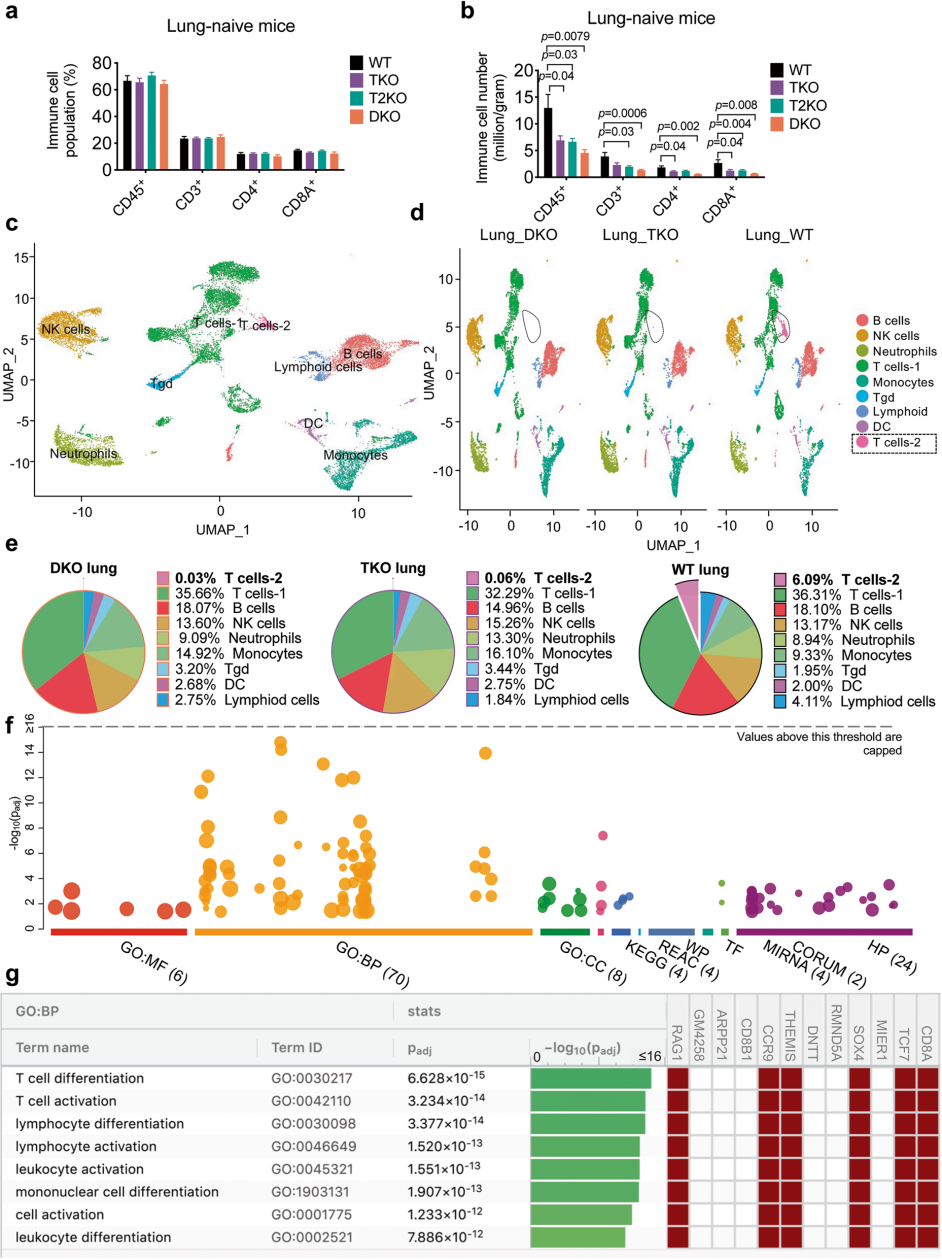
Immune cell distribution in the lung of naïve mice in the presence or absence of TIPE proteins. **a,b**, Percentage (a) and number (b) of total CD45^+^ cells in the lung of naïve mice under homeostasis state as determined by flow cytometry. *n* = 11, 13, 14 and 6 for WT, TKO, T2KO and DKO, respectively. Data were pooled from 4 independent experiments. For all graphs, data are presented as mean ± SEM, statistical significance *p* value was determined by multiple *t* test **c,d,** Single-cell RNA-seq analysis of all immune cells in the lung. UMAP clustering of all cells (c) and cells of the WT, TKO and DKO genotype (d). **e,** Pie chart showing the percentage of immune cell populations in different genotypes. **f,g,** Enrichment analysis of differentially expressed genes (f) and related pathways (g) in WT T cells-2 cluster using *GProfiler*

To test whether TIPE proteins regulate immune cell positioning in the lung at homeostasis, we digested lung samples and analyzed the immune profiles of naïve wild type (WT), *Tnfaip8*^*−/−*^ (TKO), *Tipe2*^*−/−*^ (T2KO), and *Tnfaip8*^*−/−*^ *Tipe2*^*−/−*^ double knockout (DKO) lungs. Interestingly, although immune cell subsets did not change significantly among different genotypes (Fig. 1a), the total number of CD45^+^ cells was decreased in the knockout lungs, especially DKO (Fig. 1b). The lung immune cells included lymphocytes, NK cells, and myeloid cells (Fig. 1a and Supplementary Fig. 1). Of particular interest, for the lymphocyte population, we found fewer CD3^+^CD4^+^ and CD3^+^CD8^+^ cells in the lung tissue of single knockout mice, with a further decreased number of these cells in DKO mice (Fig. 1b).

### scRNA-seq analysis reveals a unique mucosal T cell population that is dependent on TNFAIP8 and TIPE2

**Fig. 2:**
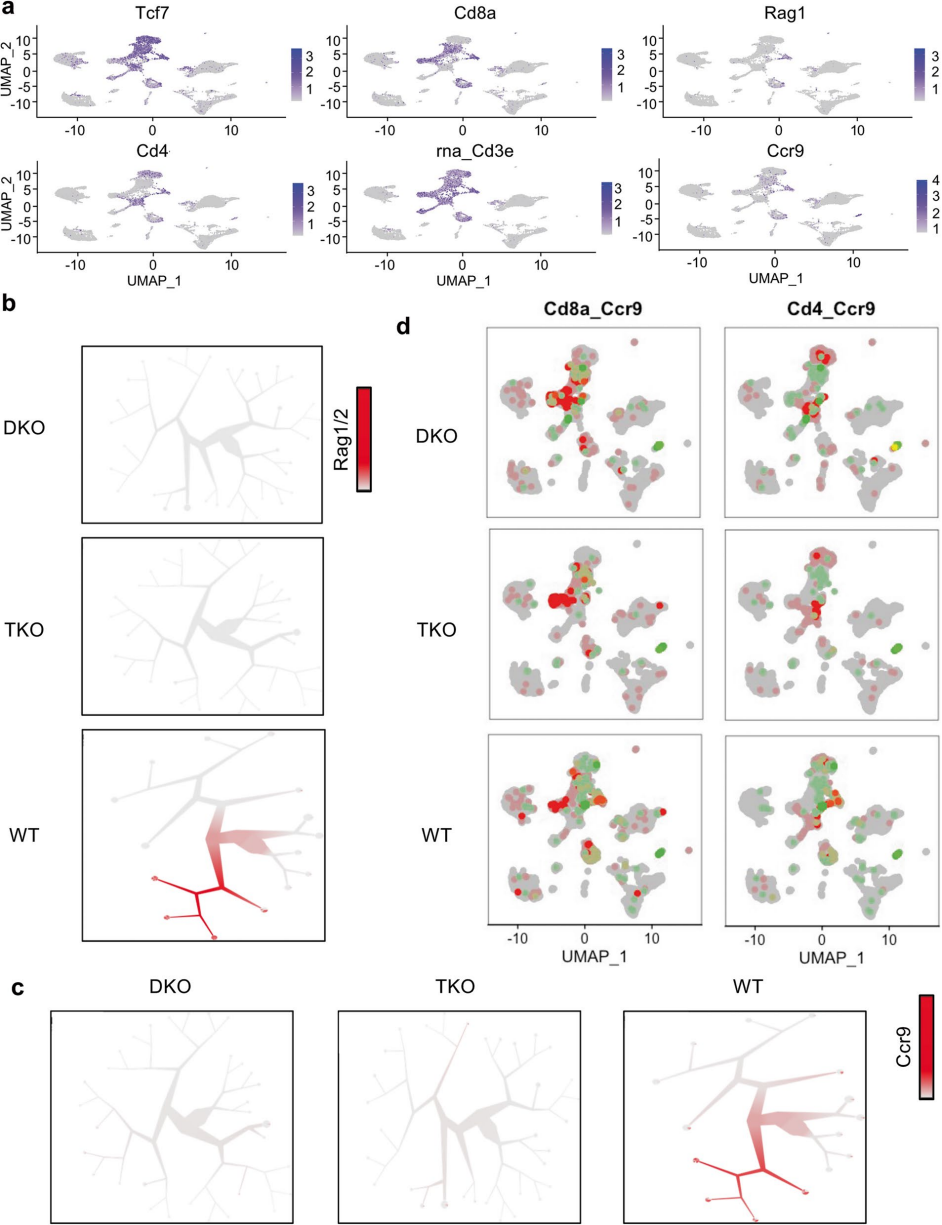
Significantly enriched genes in WT T cells-2 cluster in Seurat and TooManyCells. **a,** Feature plot showing the selected gene expression in different clusters in Seurat. **b,c,** A dendrogram representation of WT T cells showing the expression of Rag1/2 (b, bar = 0–0.5 for DKO, 0–0.3 for TKO and 0–10 for WT) and Ccr9 (c, bar = 0–12.5 for DKO and TKO, bar = 0–7 for TKO) in TooManyCells. **d,** Feature plot showing the co-expression patterns of Cd8a^+^Ccr9^+^ and Cd4^+^Ccr9^+^ in the identified clusters. Grey: All cells; Red: Cd8a^+^ (or Cd4^+^); Green: Ccr9^+^; Yellow: Cd8a^+^ (or CD4^+^) Ccr9^+^. *Cd8a*- and *Cd4*-expressing cells were in red; *Ccr9*-expressing cells were in green, and cells expressing both were in yellow

**Fig. 3:**
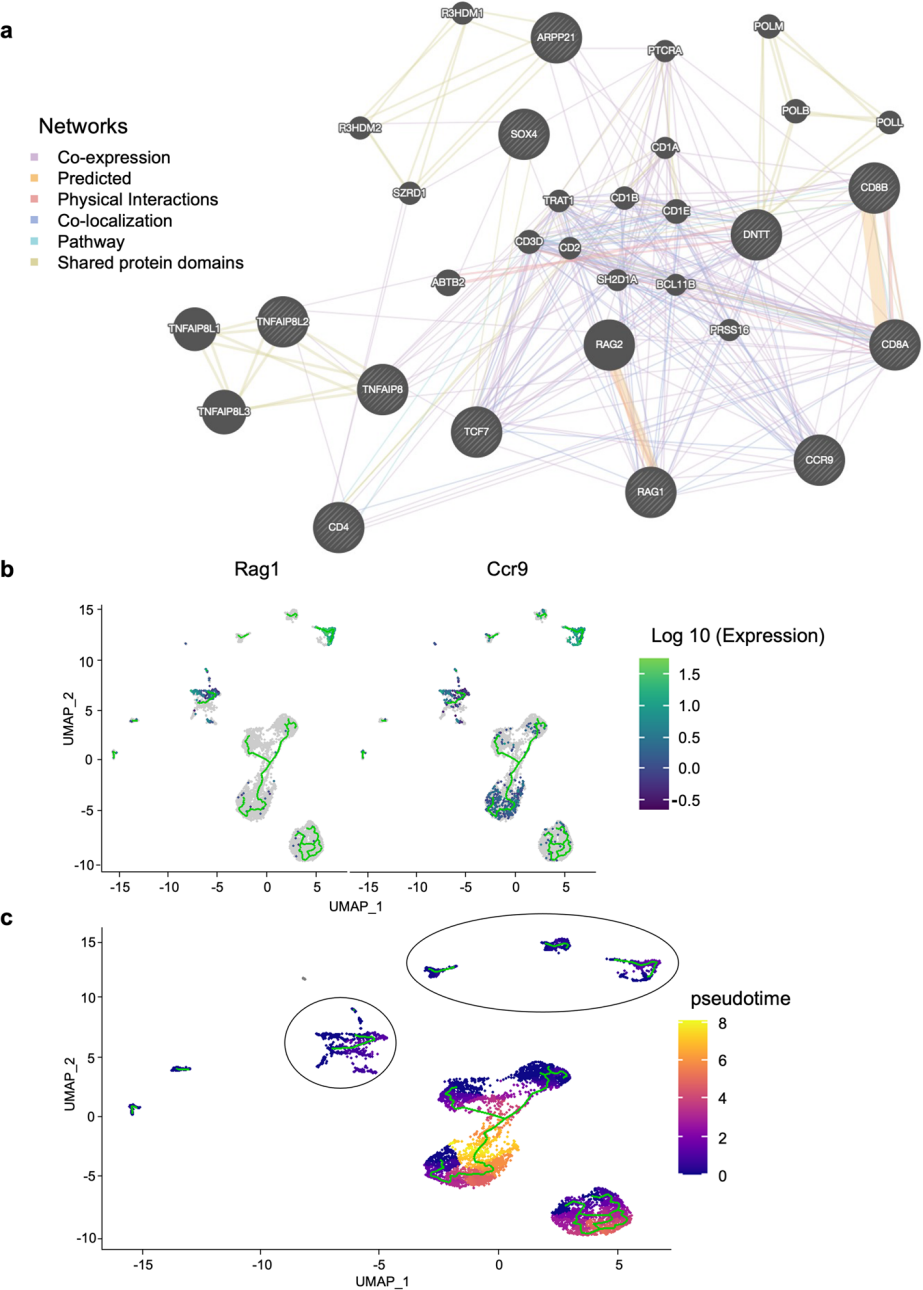
Significantly enriched genes in WT T cells-2 cluster in network analysis and Pseudotime analysis. **a,** Network analysis of significantly enriched genes in T cells-2 cluster using The GeneMANIA prediction server. **b,** Pseudotime analysis of all T cell clusters **c,** The expression of Ccr9 and Rag1

To further explore the immune cell differences among WT, TKO and DKO mice, we performed single-cell RNAseq using a 10× Genomics platform on isolated lung immune cells of age- and gender-matched mice. The expression of the top differentially expressed genes among the three groups were listed in the heatmap (Supplementary Fig. 2). Immune cells were clustered in Uniform Manifold Approximation and Projection (UMAP) using the Seurat package as shown in Fig. 1c. The clusters were then annotated into their corresponding cell types using the R Package *Single* R, which compares single-cell transcriptomes with reference transcriptomic datasets [24] (Supplementary Fig. 3a). Immune cells in the lung were identified as T cells-1, T cells-2, B cells, NK cells, neutrophils, monocytes, gamma/delta T (Tgd) cells, (other) lymphoid cells and DC (Fig. 1c-e). T cells represented one of the largest immune cell populations, and among which T cells-2 cluster was significantly decreased in TKO (0.06%) and almost completely lost in DKO (0.03%), as compared with WT group (6.09%) (Fig. 1d and e). To distinguish the T cells-2 cluster from T cells-1 cluster, we extracted the top differentially expressed genes between them and did a functional enrichment analysis using webserver g:Profiler [25]. The analysis showed that the differentially expressed genes included those important for lymphocyte (T cell) differentiation and activation (Fig. 1f), with *Rag1, Cd8a, Tcf7, Cd3e, Ccr9* (Fig. 1g) being the most differentially expressed. The global expression of these genes was plotted in Fig. 2a. *Rag1* was preferably expressed in T cells-2 with a very low expression seen in the T cells-1 cluster (Fig. 2a and b). The presence of T cell-2 in WT mice and its marked decrease in TKO and DKO mice were also confirmed by the *Too Many Cells* dendrogram analysis (Fig. 2b and c). Further analysis of CCR9 (Fig. 2c and d) is discussed in the following section. Network analysis demonstrated that *Tnfaip8* and *Tnfaip8l2* genes were linked to Tcf7-Rag1/2-Ccr9-Cd4/8 T cells (Fig. 3a). To understand the time-related expression of these clusters, we did a pseudotime analysis, which showed the *Rag1*-expressing cluster (T cells-2) as an early-stage T cell component (Fig. 3b and c). We found that although the *Rag-1* expressing T cells also expressed *Ccr9*, the expression of *Ccr9* was not exclusive to the *Rag1*-expressing T cells-2 cluster. Interestingly, the pseudotime expression graph showed decreased expression of *Rag1* as T cells further differentiate (Supplementary Fig. 3b), whereas *Ccr9,* given its ubiquitous expression in other T cells, seemed to vary in expression at different maturation stages (Fig. Supplementary Fig. 3c). Based on our single cell RNA-seq results, we believe that the T cells-2 cluster we identified represents mostly CD3^+^ TCRalpha/belta T cells (Fig. 2 and Supplementary Figs. 4 and 5).

### CCR9 is a key lymphocyte homing receptor to lung and TIPE proteins control immune cell positioning in lung mucosa

**Fig. 4:**
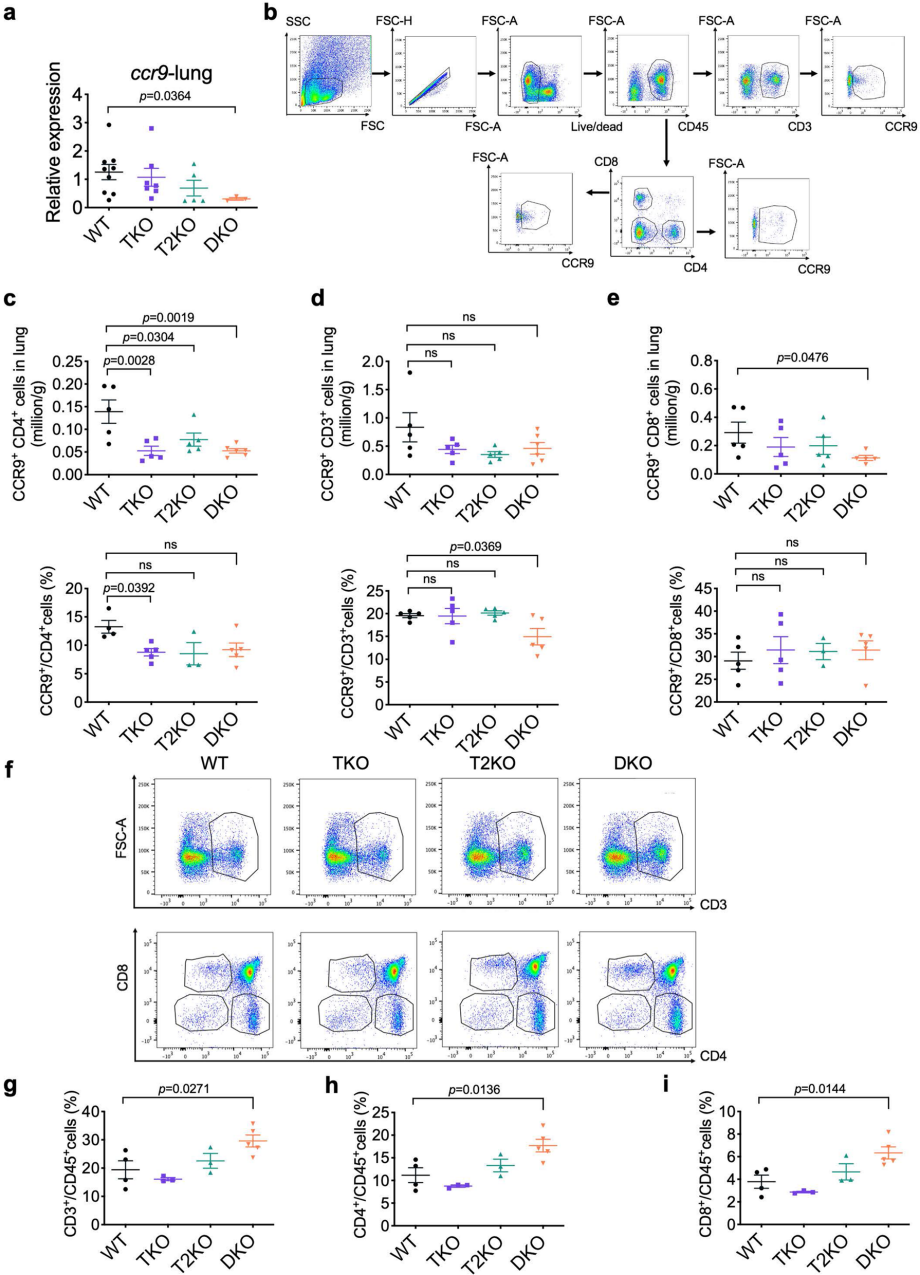
CCR9 expression in cells of lung and thymus. **a,** Relative mRNA levels of the *Ccr9* genes in the lung tissue of naïve mice. *n* = 9, 7, 5 and 3 for WT, TKO, T2KO and DKO, data were pooled from 2 to 3 independent experiments as determined by real-time PCR. **b,** Gating strategy for flow cytometry analysis of lung lymphocytes. **c-e,** Absolute numbers and percentages of CCR9^+^CD4^+^ (c), CCR9^+^CD3^+^ (d), CCR9^+^CD8^+^ (e) cells in lung CD45^+^cells. *n* = 5, 5, 5 and 6 for WT, TKO, T2KO and DKO mice. **f,** Representative flow cytometric images of CD3^+^, CD4^+^, CD8^+^ cells in CD45^+^ cells from WT, TKO, T2KO and DKO thymuses. **g-i,** The percentages of CD3^+^ (g), CD4^+^ (h), CD8^+^ (i) cells in naïve thymuses CD45^+^cells. *n* = 4, 3, 3 and 5 for WT, TKO, T2KO and DKO For all graphs, data are presented as mean ± SEM. Statistical significance *p* value was determined by Mann-Whitney test (a) or one-way ANOVA (others)

*Ccr9* is one of the most differentially-expressed genes in T cells-2 cluster compared with other clusters (Fig. 1f and g). It is a lymphocyte homing receptor for the gut [12]. We found that *Ccr9* was highly expressed in WT lymphocytes (Fig. 2a and c), which was significantly reduced in the TKO and DKO groups (Fig. 2c and d). We further validated *Ccr9* gene expression in the lung by qPCR. Compared with WT group, the expression of *Ccr9* gene was significantly reduced in the DKO group (Fig. 4a). In all KO groups, the absolute number of CCR9^+^CD4^+^ cells was significantly reduced (Fig. 4b and c) compared with WT. Additionally, there was a significant decrease in the percentage of CCR9^+^CD3^+^ cells (Fig. 4d) and the number of CCR9^+^CD8^+^ (Fig. 4e) cells in DKO group. Most T cells including CD4 and CD8 T cells and IELs, develop from hematopoietic stem cells (HSC) in the thymus and subsequently leave the thymus to enter other parts of the body [26, 27]. In DKO mice, thymic CD3^+^, CD4^+^, CD8^+^ cells (Fig. 4f-i) increased significantly suggesting that TIPE proteins may regulate thymocyte egress.

### TNFAIP8 and TIPE2 protect mice from pulmonary *Streptococcus pneumoniae* infection

**Fig. 5:**
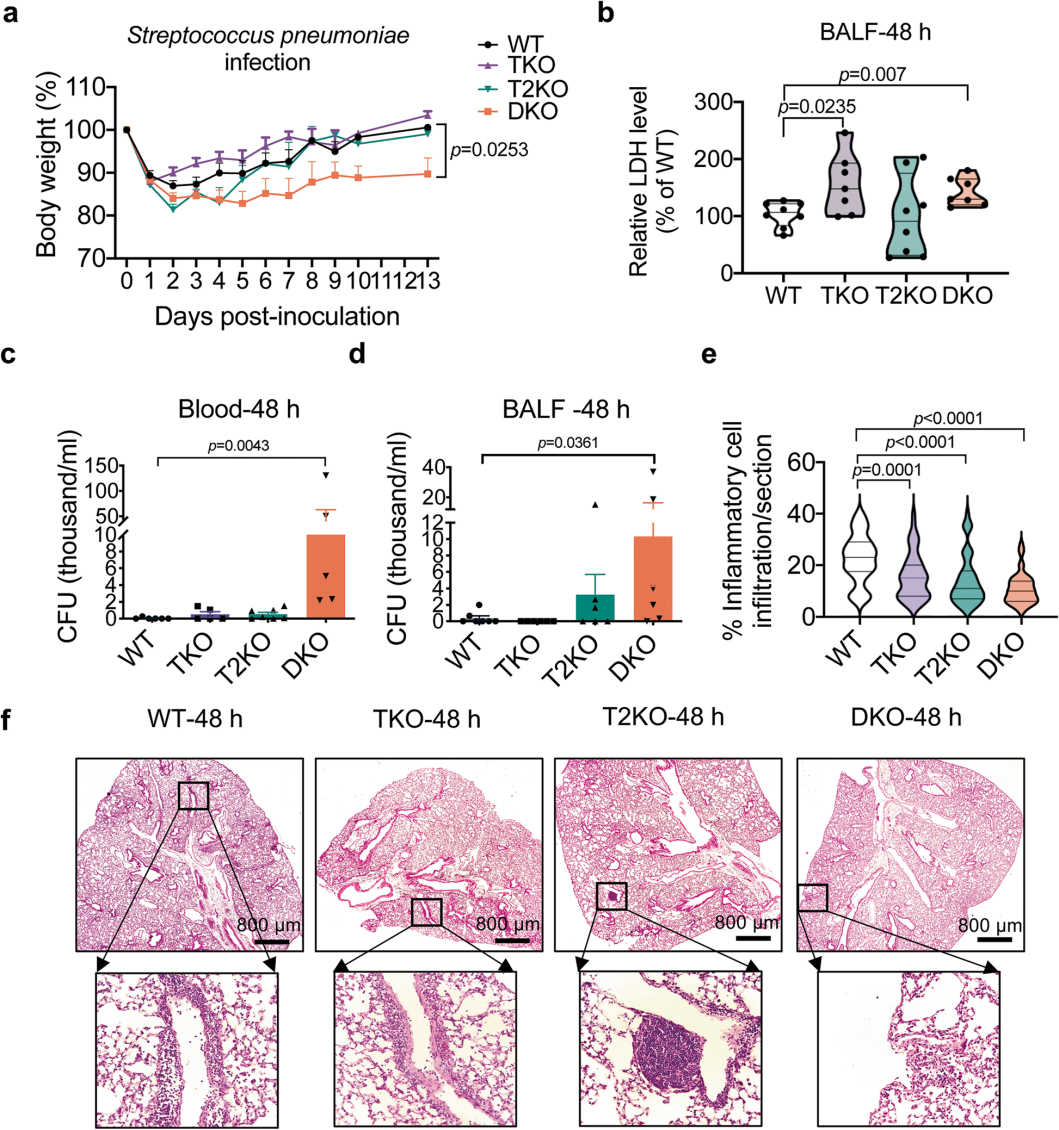
TIPE family of proteins are critical for host defense against pulmonary *Streptococcus pneumoniae* infection. **a,** Body weight of WT, TKO, T2KO and DKO mice infected with *Streptococcus pneumoniae*; *n* = 20, 17, 13 and 19 for WT, TKO, T2KO and DKO mice, pooled from four independent experiments. Statistical significance for body weight was determined by Multiple unpaired *t* tests. **b,** Relative LDH level in BALF at 48 h after *Streptococcus pneumoniae* challenge. *n* = 8, 7, 8 and 7 for WT, TKO, T2KO and DKO mice, pooled from 2 independent experiments. **c,d,** Bacterial counts in blood (c) and bronchoalveolar lavage fluid (BALF) (d) at 48 h after *Streptococcus pneumoniae* challenge. n = 5–7 mice, pooled from 2 independent experiments. **e,f,** Representative H&E staining (f) and quantification of inflammatory cell infiltration (e) of lung sections at 48 h after *Streptococcus pneumoniae* challenge. *n* = 6–8 mice in each group, pooled from 2 independent experiments, 5–10 random sections per lung were examined. Scale bars = 800 μm. Statistical significance *p* value was determined by Multiple unpaired *t* tests (a), unpaired *t* tests (b, c, d) or one-way ANOVA (e). For all graphs, data are presented as mean ± SEM

To test the consequence of TIPE deficiency on pulmonary immunity, we used a *Streptococcus pneumoniae* pulmonary infection model. In this model, mice were inoculated with the bacteria nasally, which results in infection of the lower respiratory tract and acute bacterial pneumonia [28]. *Streptococcus pneumoniae* can also migrate into the bloodstream to cause systematic infection in secondary organs (e.g. liver and spleen) [29]. Single TIPE gene KO mice had similar body weight loss (Fig. 5a) compared to WT mice, whereas DKO mice experienced significantly more weight loss (Fig. 5a). This result indicates that TNFAIP8 and TIPE2 play a redundant role in protecting against *Streptococcus pneumoniae* infection. KO mice showed a trend of increased tissue damage as determined by LDH levels from BALF (Fig. 5b). Colony formation unit (CFU) was used to monitor the severity of the infection through viable clonogenic cell number counting. Consistent with the above results, the CFUs of *Streptococcus pneumoniae* from blood (Fig. 5c) and bronchoalveolar lavage fluid (BALF, Fig. 5d) were higher in DKO than WT or single KO mice. Bacterial burden from the lung and spleen was not significantly different between genotypes (Supplementary Fig. 7 g and h). Compared with the WT group, fewer immune cells were present in DKO lungs (Fig. 5e and f) at 48h after infection.

**Fig. 6:**
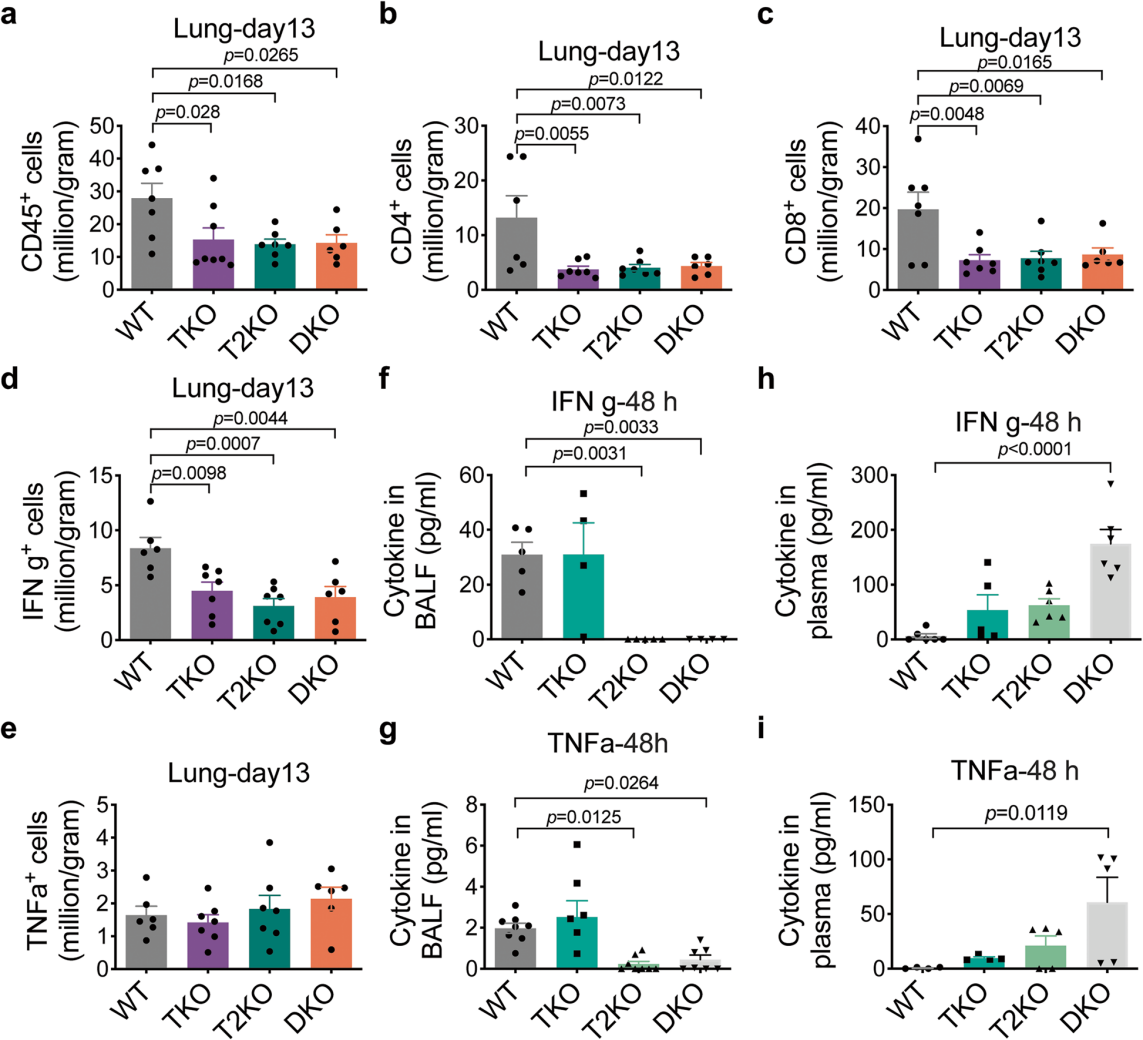
Immune cell profile of the lung after pulmonary *Streptococcus pneumoniae* infection. **a-c,** CD45^+^ (a), CD4^+^ (b) and CD8^+^ (c) cell numbers in lung at day 13 after *Streptococcus pneumoniae* challenge. n = 6–8 mice, pooled from 2 independent experiments as determined by flow cytometry. **d,e,** IFNg- (d) and TNFα-secreting cell numbers (e) in the lung at day 13 after *Streptococcus pneumoniae* challenge, as determined by flow cytometry. n = 6–8 mice, pooled from 2 independent experiments. **f,g,** IFNg (f) and TNFα (g) concentrations in BALF at 48 h after *Streptococcus pneumoniae* challenge, as determined by ELISA. n = 6–8 mice, pooled from 2 independent experiments. **h,i,** IFNg (h) and TNFα (i) concentrations in plasma at 48 h after *Streptococcus pneumoniae* challenge, as determined by ELISA. n = 6–8 mice, pooled from 2 independent experiments. Statistical significance *p* value was determined by one-way ANOVA. For all graphs, data are presented as mean ± SEM

To dissect the protective roles of TIPE proteins, we analyzed CD45^+^, CD4^+^, CD8^+^, IFNg- and TNFα-secreting cells in the lung at day 13 after infection. There were fewer CD45^+^, CD4^+^, CD8^+^, and IFNg- (but not TNFα)-secreting cells in lungs of all KO mice as compared with the WT group (Fig. 6a-e). Cytokine ELISA results further confirmed that, at 48 h after infection, the secretion of IFNg (Fig. 6f) and TNFα (Fig. 6g) in T2KO and DKO BALF was significantly reduced as compared with WT and TKO mice. Additionally, the amounts of IFNg (Fig. 6h) and TNFα (Fig. 6i) in DKO plasma were significantly higher than that in the WT plasma, likely because of more *Streptococcus pneumoniae* entering the blood (Fig. 5c) in DKO mice. Several other immune parameters tested were not affected by TIPE deficiency after infection (Supplementary Fig. 6 and 7). Therefore, in this model, the reduction in lymphocyte numbers in DKO lung mucosal site likely resulted in less bacterial-killing and more *Streptococcus pneumoniae* escape into blood circulation, ultimately causing a greater decrease of body weight.

## Discussion

These results demonstrate that TIPE proteins are regulators of T lymphocyte positioning in lung mucosa and are important for protection against bacterial infection. Our data show that without TNFAIP8 and TIPE2, T lymphocytes do not efficiently home to the lung, and thus lead to the reduced numbers in the lung. As a result, in DKO mice, more pathogens enter blood circulation from the mucosa and cause systematic infection, resulting in a significantly decreased body weight.

CCR9 plays an essential role in recruiting IEL to the small intestine [30, 31], and engages in early mucosal T cell development [12]. CCR9 expression is restricted to CD4^+^ and CD8^+^ T lymphocytes in the small intestine, as opposed to other organs such as tonsil, liver, and skin, etc. [31]. In addition to directing lymphocyte trafficking to the small intestine, CCR9 participates in tumor immunity and tumor development [32–36]. Similar to gut, the lung is one of the largest mucosal sites, and studies have shown an important role for CCR9 in promoting migration and suppressing apoptosis of lung cancer cells [35, 37]. Therefore, it’s likely that CCR9 could mediate lymphocyte homing to the lung. Consistent with this view, we found that CCR9-expressing lymphocytes accounted for more than 30% and 12% of total lung CD8^+^ and CD4^+^ cells, respectively.

Aerosol (inhalation) route is one of the most common ways of microorganism infection. Upon inhalation of contaminated air, pathogens such as *Streptococcus pneumoniae* come into contact with the respective mucosal epithelium, where a large number of resident innate and adaptive immune cells reside. Only those pathogens that have escaped from the mucosal immune attacks can transmigrate through the epithelial barrier and enter the blood stream, where they are greeted by the systemic immune defense mechanisms. Our previous work demonstrated that TKO [22] and T2KO [23] mice are both resistant to intravenous injection (iv) of *L. monocytogenes* due to enhanced immune cell effector function and/or altered liver cell response to listeria infection. In the intravenous model, pathogens were injected into mice at equal amount, bypassing the mucosal immune system. In the mucosal model studied here, we tested the natural infection route and discovered the protective role of TIPE proteins against pulmonary pathogens. In DKO mice, more pathogens entered the blood circulation, likely due to the reduced number of lymphocytes at the mucosa, resulting in more severe infection as compared with WT mice.

In summary, we discovered that TIPE proteins are required for deployment of T lymphocytes to the lung to protect against local infection. Therefore, TIPE-based strategies could help develop mucosal vaccines or treat inflammatory diseases of the lung.

## Materials and methods

### Mice

*Tnfaip8* ^*−/−*^ (TKO) and *Tipe2*^*−/−*^ (T2KO) C57BL/6 mice were generated as described [21, 38]. The double-knockout (DKO) mice were generated by crossing TKO with T2KO mice which were generated as described previously [17]. Groups of 8–12 weeks-old female WT and knockout mice were used for infection. All mice were housed at the University of Pennsylvania Animal Care Facilities under pathogen-free conditions. All animal protocols and were preapproved by the use committee and animal care of University of Pennsylvania.

### Mucosal cell preparation

After blood was taken from cardiac puncture, mice were perfused with PBS transcardially, and several organs (lung, thymus, spleen, etc.) were collected. Lung tissue was collected, measured, cut into small pieces and digested in lung digestion buffer at 37 °C water bath for 30 mins. The digestion buffer contains 10% DMEM, 0.1% β-mercaptoethanol, 1% pen-strep, 0.15 mg/ml DNAase I (Roche-10104159001 Sigma Millipore, Burlington, MA), 3 mg/ml Collagenase IV (17140019 Thermo Fisher, Waltham, MA). The digested tissues were filtered through 70 μm cell strainers (BD Biosciences, San Jose, CA). After red cell lysis by ACK buffer, all cells were counted and the total number of cells per gram tissue was calculated.

### Flow cytometry

For surface marker staining, cells were first incubated with Fc blockers (1:1000) for 15 min at room temperature, followed by Zombie Aqua Fixable Viability kit reagents (423101 Biolegend, San Diego, CA) in PBS for 15 min at RT. Then cells were stained with a cocktail of antibodies in FACS buffer (1% BSA in PBS). All antibodies for surface markers were acquired from Biolegend and diluted at 1:300 for staining, unless specified otherwise. For intracellular staining, cells were seeded at 2 million per 100 μl medium in round bottom plate and stimulated with 10 ng/ml PMA and 0.75 μl /ml inomycin, plus Brefeldin A (1:1000) and Golgi stop (1:1000) for 5 h. Cells were washed, stained with Zombie Aqua Fixable Viability kit and fixed by 1% PFA in FACS buffer for 10 min. Cells were washed twice with FACS buffer, stained with antibodies to surface markers and followed by permeabilization with 0.1% Triton in PBS. Finally, cells were stained with antibodies to intracellular proteins, and analyzed by flow cytometer.

### Single-cell RNA-seq analysis of lung immune cells

Eight to 10-week-old female naïve mice of WT, TKO and DKO genotypes (three mice per group) were perfused with PBS, and lungs were collected. Percoll was used to enrich immune cells. Briefly, lung samples were suspended in 44% percoll (GE17–0891-01, Sigma Millipore), and then 67% percoll was added slowly and carefully to the bottom of 50 ml tubes. Cells were then spun at 1000 g, 10 mins at 4 °C, and cells at the interface of the percoll gradient were collected and stained with anti-CD45-FITC. Live CD45^+^ cells were sorted and subjected to single-cell RNAseq. The cell viability was maintained at more than 90% and no less than 10,000 cells per group were loaded to generate libraries.

Gene expression profiles in each genotype were analyzed using Loupe Browser (10 × Genomics) [39]. Seurat version 3.2.2 was used for cluster identification in scRNAseq datasets [40, 41]. The raw counts data were read in R and normalized by log-transformation. Cells with a mitochondrial ratio above 0.2 were removed. Using Seurat’s “FindIntegrationAnchors” and “IntegrateData” functions, cells from WT and KO mice were integrated into a single analysis. For UMAP (Uniform Manifold Approximation and Projection) dimension reduction and clustering analysis, we used the first 30 principal components. SingleR [24] was used to identify the cell types of the identified clusters. Cluster-specific markers were determined using the “FindAllMarkers” function and used in GProfiler [25] for enrichment analysis and their expression was visualized in R using the “Feature Plot” function in Seurat. Violin plots were generated using Seurat’s “VlnPlot” function. The Monocle3 package was used for pseudotime analysis and to generate the pseudotime expression graphs [42, 43]. The integrated preprocessed Seurat object was normalized, clustered, and visualized using UMAP for dimensionality reduction. TooManyCells was used to visualize transcriptionally similar cells and generate dendrograms [44]. Gene sets functions were predicted by The GeneMANIA prediction server [45].

### *Streptococcus pneumoniae* strain and infection with TIPE deficient mice

*Streptococcus pneumoniae* strain TIGR4 (serotype 4) was a kind gift from Dr. Hao Shen (Department of Microbiology, University of Pennsylvania, Philadelphia, PA). This strain was grown at 37°C with 5% CO_2_ in Tryptic soy medium or agar plates (BD Biosciences) as previously described [28]. For lung infection, mice were anesthetized intraperitoneally with 100 μl ketamine /xylazine (100 mg/3.8 mg kg) and intranasally infected with 30 μl of bacterial suspensions (1–2 × 10^7 CFU/ mouse). Body weight and clinical signs of morbidity were monitored daily.

### Gross pathology and histopathology

In the infection model, body weight was monitored. At the end of the experiment, blood, organs, BALF were collected for further analysis. Lung samples (pulmonary infection) were fixed with 10% formalin and processed for paraffin sections. For histologic examination, lung sections were stained with hematoxylin and eosin (H&E staining), and lung immune cell numbers were counted in a double-blinded manner, and presented by the percentage of immune cells.

### Bronchoalveolar lavage fluid (BALF) cells collection

In the pulmonary infection model, blood was taken from cardiac puncture and the lung was washed with sterile 0.8 ml lavage buffer (PBS plus 100 μM EDTA) twice to get BALF. BALF was centrifuged at 300 g for 5 min at 4 °C, and supernatants were stored at − 80 °C. Pelleted cells in the BALF were counted and analyzed by flow cytometry to determine immune cell profile. Total protein and LDH levels were determined by BCA kit (PI23225, Thermo Fisher) and CyQuant LDH Cytotoxicity Assay kit (Thermo Fisher) according to manuals, respectively.

### Bacterial enumeration

Colony formation unit (CFU) counts were determined at the indicated time points by plating serial dilutions of culture supernatants onto agar plates. In the pulmonary model, after the spread of 100 μl Catalase (LS001896, Worthington, NJ) on Tryptic soy agar plates (90002–706, Biosciences), CFUs of blood, BALF, smashed infected organs were determined by serial dilutions in triplicates. Bacterial colonies were counted after 24 h at 37 °C with 5% CO_2_.

### Cytokine detection by ELISA

Plasma was collected from mice after infection and kept at − 80 °C. Antibodies used in ELISA were purchased from eBioscience, Biolegend or BD Pharmingen including standard protein, purified, and biotinylated antibodies. Paired antibodies were used to perform ELISA for corresponding cytokines per the manufacturer’s recommendations.

### RNA isolation and quantitative real-time PCR

Tissue RNA was extracted using the RNeasy Mini Kit (QIAGEN, Germantown, MD) per manufacturer’s instructions. Reverse transcription was carried out with a high-capacity reverse transcription kit (Applied Biosystems, Foster City, CA) per manual. Real-time PCR was performed using the Fast SYBR Green PCR Master Mix (Applied Biosystems) in Applied Biosystems 7500 Real-Time PCR System. Relative fold change was calculated by the ΔΔCT method with 18s ribosomal RNA as an internal control. Primers were synthesized by Integrated DNA Technologies (Coralville, Iowa) as sequence follows: *18s* (5′-GTA ACC CGT TGA ACC CCA TT-3’and 5′-CCA TCC AAT CGG TAG TAG CG-3′); *Ccr9* [46] (5′-CAA TCT GGG ATG AGC CTA AAC AAC 3′ and 5′-ACC AAA AAC CAA CTG CTG CG-3′).

## Supplementary Information

**Additional file 1: Supplementary Fig. 1.** Immune cell profile of naïve mouse lung. **Supplementary Fig. 2.** Top differentially expressed genes of naïve lung immune cells among three genotypes of mice. **Supplementary Fig. 3.** Overall view of immune cell clusters and differentially expressed genes. **Supplementary Fig. 4.** TCR and NKT marker gene expression in lung immune cells as determined by scRNAseq. **Supplementary Fig. 5.** MAIT cell marker gene expression in lung immune cells as determined by scRNA-seq. **Supplementary Fig. 6.** Immune cell profile of lung after *Streptococcus pneumoniae* challenge. **Supplementary Fig. 7.** Cytokine level and bacterial counts at 48 h after *Streptococcus pneumoniae* infection.

## Data Availability

The data discussed in this publication have been deposited in NCBI’s Gene Expression Omnibus [47] and are accessible through GEO Series accession number GSE185256 (https://www.ncbi.nlm.nih.gov/geo/query/acc.cgi?acc=GSE185256). The datasets generated during and/or analyses during the current study are available from the corresponding author on reasonable request.

## Abbreviations

BALF: Bronchoalveolar lavage fluid
CCL25: C-C Motif Chemokine Ligand
CCR9: C-C Motif Chemokine Receptor 9
CFU: Colony formation unit
DKO: *Tnfaip8* ^*−/−*^ *Tipe2*^*−/−*^ double knockout
H&E staining: Hematoxylin and Eosin staining
scRNAseq: Single cell RNA sequencing
*Sp.*: *Streptococcus pneumoniae*
Tgd: gamma/delta T cells
TIPE/TNFAIP8: tumor necrosis factor-α-induced protein 8
TIPE1: TNFAIP8L1/ tumor necrosis factor-α-induced protein 8 like1
TIPE2: TNFAIP8L2/ tumor necrosis factor-α-induced protein 8 like2
TIPE3: TNFAIP8L3/ tumor necrosis factor-α-induced protein 8 like3
UMAP: Uniform Manifold Approximation and Projection
