# Supplementary material for: TIPE polarity proteins are required for mucosal deployment of T lymphocytes and mucosal defense against bacterial infection
Source: Mol Biomed. 2021 Dec 23;2:41. doi: 10.1186/s43556-021-00059-8 (PMC8695405; doi:10.1186/s43556-021-00059-8)
Supplement: Supplementary file 1 — Additional file 1: Supplementary Fig. 1. Immune cell profile of naïve mouse lung. Supplementary Fig. 2. Top differentially expressed genes of naïve lung immune cells among three genotypes of mice. Supplementary Fig. 3. Overall view of immune cell clusters and differentially expressed genes. Supplementary Fig. 4. TCR and NKT marker gene expression in lung immune cells as determined by scRNAseq. Supplementary Fig. 5. MAIT cell marker gene expression in lung immune cells as determined by scRNA-seq. Supplementary Fig. 6. Immune cell profile of lung after Streptococcus pneumoniae challenge. Supplementary Fig. 7. Cytokine level and bacterial counts at 48 h after Streptococcus pneumoniae infection. [file 43556_2021_59_MOESM1_ESM.docx]

**Supplementary Information**

**TIPE polarity proteins are required for mucosal** **deployment of T lymphocytes and mucosal defense against bacterial infection**

Mingyue Li^1,2*^, Mayassa J. Bou-Dargham^1^, Jiyeon Yu^1^, Zienab Etwebi^1^, Honghong Sun^1*^, Youhai H. Chen^1,3*^

^1^Department of Pathology and Laboratory Medicine, Perelman School of Medicine, University of Pennsylvania, Philadelphia, PA, USA.

^2^The Wistar Institute, Philadelphia, PA19104, USA

^3^Faculty of Pharmaceutical Sciences, CAS Shenzhen Institute of Advanced Technology, Shenzhen, China.

*Corresponding authors:

Dr. Youhai H. Chen, 713 Stellar-Chance Laboratories, 422 Curie Blvd. Philadelphia, PA 19104,

215-898-4671, [yhc@pennmedicine.upenn.edu](mailto:yhc@pennmedicine.upenn.edu)

Dr. Mingyue Li, 712 Stellar-Chance Laboratories, 422 Curie Blvd., Philadelphia, PA 19104,

215-898-7962, [Mingyue.Li@pennmedicine.upenn.edu](mailto:Mingyue.Li@pennmedicine.upenn.edu)

Dr. Honghong Sun, 713 Stellar-Chance Laboratories, 422 Curie Blvd., Philadelphia, PA 19104,

215-898-7962, [hsun2@pennmedicine.upenn.edu](mailto:hsun2@pennmedicine.upenn.edu)

**Supplementary Fig. 1. Immune cell profile of naïve mouse lung.**

**a,** Gating strategy of flow cytometry for the analysis of lung cells.

**b,c,** Numbers (b) and percentages (c) of the indicated lung immune cells of naïve mice as determined by flow cytometry. n=6-14 mice, pooled from 4 independent experiments.

**Supplementary Fig. 2.** **Top differentially expressed genes of naïve lung immune cells among three genotypes of mice.** The analysis was done using Loupe Browser (from 10 × Genomics) with heatmap presented on a log2 scale.

**Supplementary Fig. 3. Overall view of immune cell clusters and differentially expressed genes.**

**a,** SingleR identification of immune cell types corresponding to each cluster.

**b,c,** Pseudotime expression graph representative of *Rag1* (b) and *Ccr9* (c) at different developmental and maturation points (pseudotime analysis).

**Supplementary Fig. 4. TCR and NKT marker gene expression in lung immune cells as determined by scRNAseq.**

a,b, Feature plots showing the T cell receptor alpha constant gene (Trac) (a) and T cell receptor delta constant gene (Trdc) (b) expression by different clusters of cells in Seurat.

c, Feature plots showing the NKT marker gene expression by different clusters of cells in Seurat.

**Supplementary Fig. 5. MAIT cell marker gene expression in lung immune cells as determined by scRNA-seq.**

Feature plots showing MAIT marker gene expression in different cell clusters defined in Seurat.

**Supplementary Fig. 6. Immune cell profile of lung after** ***Streptococcus pneumoniae* challenge.**

**a,b,** Cell percentage (a) and number (b) in the spleen at 48 h after *Streptococcus pneumoniae* challenge, as determined by flow cytometry. n=6-7 mice pooled from 2 independent experiments.

**c,d,** Cell percentage (c) and number (d) in the BALF at 48 h after *Streptococcus pneumoniae* challenge, as determined by flow cytometry. n=6-7 mice pooled from 2 independent experiments.

**e,f,** Cell percentage (e) and number (f) in the lung at 48 h after *Streptococcus pneumoniae* challenge, as determined by flow cytometry. n=6-7 mice pooled from 2 independent experiments.

**g,h,** Total number of NK1.1^+^ (g) and Gr1^+^ (h) cells in the lung at day 13 after *Streptococcus pneumoniae* challenge, as determined by flow cytometry. n=6-7 mice pooled from 2 independent experiments.

**Supplementary Fig. 7. Cytokine level and bacterial counts at 48 h after *Streptococcus pneumoniae* infection****.**

**a,b,** IL6 concentrations in plasma (a) and BALF (b) after infection, as determined by ELISA. n=6-7 mice pooled from 2 independent experiments.

**c,d,** IL17 concentrations in plasma (c) and BALF (d) after infection, as determined by ELISA. n=6-7 mice pooled from 2 independent experiments.

**e,f,** IL1b concentrations in plasma (e) and BALF (f) after infection, as determined by ELISA. n=6-7 mice pooled from 2 independent experiments.

**g,h,** Bacterial counts in lung (g) and spleen (h) at 48 h after infection. n=6-7 mice, pooled from 2 independent experiments.
